# Identification of Cell-Free Circulating MicroRNAs for the Detection of Early Breast Cancer and Molecular Subtyping

**DOI:** 10.1155/2019/8393769

**Published:** 2019-08-08

**Authors:** Karen C. B. Souza, Adriane F. Evangelista, Letícia F. Leal, Cristiano P. Souza, René A. Vieira, Rhafaela L. Causin, A. C. Neuber, Daniele P. Pessoa, Geraldo A. S. Passos, Rui M. V. Reis, Marcia M. C. Marques

**Affiliations:** ^1^Molecular Oncology Research Center, Barretos Cancer Hospital, Barretos, Brazil; ^2^Department of Clinical Oncology, Barretos Cancer Hospital, Barretos, São Paulo, Brazil; ^3^Department of Mastology and Breast Reconstruction, Barretos Cancer Hospital, Barretos, Brazil; ^4^Tumor Biobank, Barretos Cancer Hospital, Barretos, São Paulo, Brazil; ^5^Department of Basic and Oral Biology, School of Dentistry of Ribeirão Preto, University of São Paulo, Brazil; ^6^Life and Health Sciences Research Institute (ICVS), Health Sciences School, University of Minho, Braga, Portugal; ^7^ICVS/3B's-PT Government Associate Laboratory, Braga/Guimarães, Portugal; ^8^Barretos School of Health Sciences, FACISB, Barretos, São Paulo, Brazil

## Abstract

Early detection is crucial for achieving a reduction in breast cancer mortality. Analysis of circulating cell-free microRNAs present in the serum of cancer patients has emerged as a promising new noninvasive biomarker for early detection of tumors and for predicting their molecular classifications. The rationale for this study was to identify subtype-specific molecular profiles of cell-free microRNAs for early detection of breast cancer in serum. Fifty-four early-stage breast cancers with 27 age-matched controls were selected for circulating microRNAs evaluation in the serum. The 54 cases were molecularly classified (luminal A, luminal B, luminal B Her2 positive, Her-2, triple negative). NanoString platform was used for digital detection and quantitation of 800 tagged microRNA probes and comparing the overall differences in serum microRNA expression from breast cancer cases with controls. We identified the 42 most significant (P ≤ 0.05, 1.5-fold) differentially expressed circulating microRNAs in each molecular subtype for further study. Of these microRNAs, 19 were significantly differentially expressed in patients presenting with luminal A, eight in the luminal B, ten in luminal B HER 2 positive, and four in the HER2 enriched subtype. AUC is high with suitable sensitivity and specificity. For the triple negative subtype miR-25-3p had the best accuracy. Predictive analysis of the mRNA targets suggests they encode proteins involved in molecular pathways such as cell adhesion, migration, and proliferation. This study identified subtype-specific molecular profiles of cell-free microRNAs suitable for early detection of breast cancer selected by comparison to the microRNA profile in serum for female controls without apparent risk of breast cancer. This molecular profile should be validated using larger cohort studies to confirm the potential of these miRNA for future use as early detection biomarkers that could avoid unnecessary biopsy in patients with a suspicion of breast cancer.

## 1. Introduction

Breast cancer is the most common female cancer in the world with an estimated 1.67 million new cases diagnosed worldwide in 2012 [1]. Both clinically and biologically breast cancer is a highly heterogeneous and guidelines provided by AJCC 7th Edition Staging for Breast suggest using a classification based on five molecular subtypes: luminal A, luminal B, luminal B HER2 positive, HER2-enriched, and triple negative [2]. The extent of disease at diagnosis is strongly associated with prognosis, so that efficient and noninvasive methods for early detection of initial stage disease are key for successful treatment and improving survival [3].

Mammography is currently the best method for early detection of breast cancer, but it has some limitations due to the high number false positives and the unnecessary stress that these diagnostic errors can cause [4, 5]. Biopsy represents the gold-standard procedure for definitive diagnosis, although this procedure is invasive and may also be painful. New multigene profiling panels for breast cancer are now available, such as Oncotype DX (Genomic Healthy, USA) MammaPrint (Agendia, Netherlands) and Prosigna/PAM50 (NanoString, USA); however, these assays are designed for evaluating the risk of tumor recurrence and not suited for early cancer detection [6]. In fact, there is a critical shortage of noninvasive methods based on diagnostically sensitive and specific breast cancer biomarkers suitable for both early detection and subtype classification of tumors [7].

Liquid biopsies, such as blood samples, are less invasive and easier to obtain compared to a tissue-based biopsy. For a number of human tumors, including breast cancer, biomarker analysis of circulating microRNAs (miRNA) from serum is one of the most effective noninvasive for diagnoses and evaluation of prognosis in different diseases [8]. The extensive stability of miRNAs in peripheral blood and other body fluids together with the relative ease of detection and evaluation makes circulating miRNA ideal biomarkers to be used as liquid biopsies [9]. Moreover, there is increasing evidence that malignant mammary epithelial cells can release miRNAs into peripheral blood so that the molecular profiling of these miRNAs is an opportunity to develop new liquid biopsies for early breast cancer detection and evaluation [10, 11]. Recently our group identified two circulating miRNAs as potential tumor suppressors in invasive breast cancer [12].

In breast cancer, several miRNAs have already been reported as potential biomarkers of metastasis, recurrence, prognosis, or response to therapy [13, 14]. Examples include miR-155 that is upregulated in breast cancer [15]. Another study showed that circulating levels of miR-195 were elevated in women with breast cancer (stage I-IV) in comparison to healthy women [16]. However, at the present time, few studies have found significantly altered miRNAs biomarkers that are suitable for use in early diagnosis and detection of breast cancer.

In this study, we applied multiplexed gene expression analysis using nCounter® Technology (NanoString Technologies, Seattle, WA, EUA) to identify miRNAs in liquid biopsy samples from early-stage breast cancer patients. We present analyses of 42 clinically relevant circulating, differentially expressed miRNAs in the serum of 54 Brazilian breast cancer patients. From these miRNAs, we selected a subset of new biomarkers capable of distinguishing female breast cancer patients from matched control of healthy women without risk of this type of cancer.

## 2. Materials and Methods

### 2.1. Study Design and Patients

This is case-control study with retrospective collection of biological samples and clinical data. The early-stage (CS I and II) cases (n=54) were selected from a bigger series of breast cancer patients diagnosed at Barretos Cancer Hospital (BCH), having the following features: age range 40-69 years old; no breast cancer recurrence; absence of family history/MIRIAD >10%; confirmation of tumor stage and molecular subtype; and availability of serum prior to chemotherapy or hormone therapy. Breast cancer cases included were classified by molecular subtype according to St. Gallen International Expert Consensus on the Primary Therapy of Early Breast Cancer 2011.

The selected cases were matched to 27 controls by age (± 3 years). These controls were healthy women that underwent mammography on the Prevention Department of BCH, whose Gail Risk model was less than 1.66, mammography result was BIRADS 1 or 2 and had blood collected.

All biological samples were retrieved from Barretos Cancer Hospital Tumor Biobank. This study was approved by the Ethics Committee of Barretos Cancer Hospital (Protocol n°1212/2016), in accordance with the Declaration of Helsinki.

### 2.2. RNA Isolation from Serum Samples

Total RNA isolation was recovered from 400uL of serum obtained from cases and controls by miRNeasy Serum/Plasma Kit, including RNase-Free DNase steps (Qiagen, Gaithersburg, MD, USA). RNA quantification was performed using the NanoDrop N-100 spectrophotometer (NannoDrop Products, Wilmington, DE).

### 2.3. NanoString nCounter® System Assays

The miRNA expression analysis was performed using the nCounter® Human v3 miRNA Expression panel employing the nCounter® Analysis System (NanoString Technologies, Seattle, USA). Briefly, around 100 ng total RNA was preprocessed Tags ligation followed by hybridization with the Reporter CodeSet and Capture ProbeSet (nCounter® Human v3 miRNA Expression Assay). Samples were processed using the NanoString PrepStation and immobilized into the nCounter cartridge, which was placed into the nCounter® Digital Analyzer for image capture (280 fields of view) and data acquisition. Normalization was performed using standard procedures established by Markowitz et al., using the Aromalight package (Bioconductor) in R environment.

### 2.4. miRNA Target Prediction

Target prediction was performed by miRDIP (microRNA Data Integration Portal: http://ophid.utoronto.ca/mirDIP/). The target genes were independently selected by five algorithms (DIANA, RNA22, TargetScan, microrna.org, and RNAHybrid), using some selection criteria of presence in at least four algorithms. We only considered the top 1% of target genes, including those that had already been identified by the Cancer Gene Index data (NCI) as being involved in breast cancer. To further determine how the selected genes were associated with breast cancer and the molecular pathways that were related to these genes, we used the plugin ReactomeFI on Cytoscape (Version 3.6.0, Seattle, WA, USA). Molecular pathways were selected considering p value lower than 0.01 and pathways that included at least three genes. The interaction network was performed by Cytoscape [13].

### 2.5. Statistical Analysis

Statistical analyses were performed considering the normal distribution of samples. Student's t-test was performed, using the Bioconductor multtest package. Fold-change estimation, area under (AUC) the Operating Characteristic Curve (ROC), sensitivity and specificity analysis were performed to determine the accuracy of differentially expressed miRNAs. The ROC curve analysis was performed using the ROCR package (Bioconductor) in R program. All images resulting from this analysis were generated from the ggplot2 and ComplexHeatmaps packages (Bioconductor).

## 3. Results

### 3.1. Study Population

The clinicopathological features of the 54 patients with early-stage breast cancer (cases) are summarized in Table 1. The median age of early-stage breast cancer cases was 54.6 years old (range 41-69 years). The control group (n=27) was matched with the early-stage breast cancer cases by age (± 3 years) and the median age was 54.3 years old (range 42-67 years).

### 3.2. Identification of Differentially Expressed miRNAs in Breast Cancer Cases

All cases were stratified according clinical stage I and II and by molecular subtypes (luminal A, luminal B, luminal B Her2 positive, Her-2, and triple negative). This stratification was employed for specifically distinguishing miRNAs biomarkers from the cases and controls for early detection of breast cancer.

**Figure 1:**
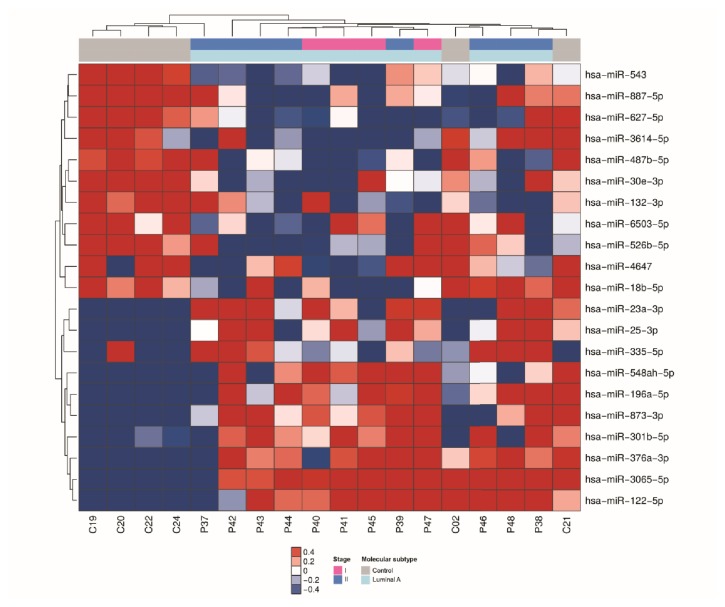
miRNAs differentially expressed in serum samples of patients with luminal A breast cancer. Heatmap demonstrating the differentially expressed miRNAs found in the serum of luminal A breast cancer patients compared with healthy women.

Of the 800 miRNAs determined by NanoString Technology, 21 had significant differential expression (P ≤ 0.05, 1.5-fold) in the luminal A subtype comprising 11 miRNAs that were downregulated and 10 miRNA that were upregulated in serum of breast cancer cases in comparison with serum from the matched healthy controls (Figure 1).

**Figure 2:**
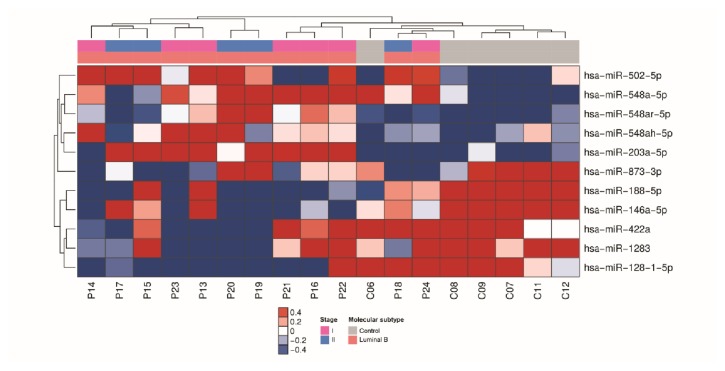
miRNAs differentially expressed in serum samples of patients with luminal B breast cancer. Heatmap demonstrating the differentially expressed miRNAs found in the serum of luminal B breast cancer patients compared with healthy women.

For luminal B subtype, 11 miRNAs had significant differential expression (P ≤ 0.05, 1.5-fold), including 6 miRNAs that were downregulated and 5 miRNAs that were upregulated in serum of cases with breast cancer in comparison with the matched healthy controls (Figure 2).

**Figure 3:**
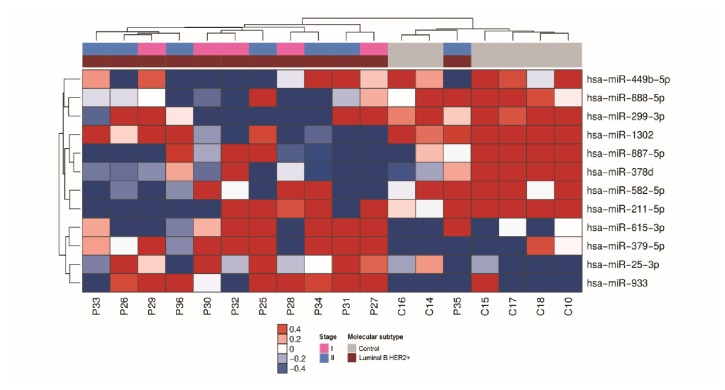
miRNAs differentially expressed in serum samples of patients with luminal B HER2 positive breast cancer. Heatmap demonstrating the differentially expressed miRNAs found in the serum of luminal B HER 2 positive breast cancer patients compared with healthy women.

For luminal B HER2 positive subtype, 12 miRNAs had significant differential expression (P ≤ 0.05, 1.5-fold), including 8 miRNAs that were downregulated and 4 miRNAs that were upregulated in serum of cases with breast cancer in comparison with matched healthy controls (Figure 3).

**Figure 4:**
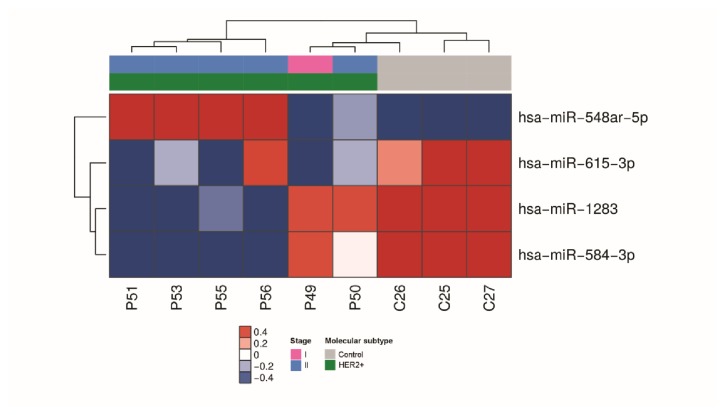
miRNAs differentially expressed in serum samples of patients with HER2-enriched breast cancer. Heatmap demonstrating the differentially expressed miRNAs found in the serum of HER2-enriched breast cancer patients compared with healthy women.

For HER 2-enriched subtype, 4 miRNAs had significant differential expression (P ≤ 0.05, 1.5-fold), including 3 miRNAs that were downregulated and one miRNA that was upregulated in serum of breast cancer cases compared with matched healthy controls (Figure 4).

**Figure 5:**
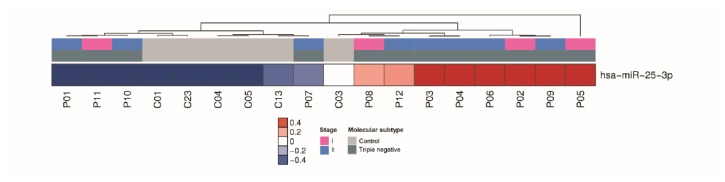
miRNAs differentially expressed in serum samples of patients with triple negative breast cancer. Heatmap demonstrating the differentially expressed miRNAs found in the serum of triple negative breast cancer patients compared with healthy women.

For triple negative subtype, only miR-25-3p was upregulated (P ≤ 0.05, 1.5-fold) in serum of the cases with breast cancer in comparison with matched healthy controls (Figure 5).

### 3.3. Evaluation of Circulating miRNAs as Biomarkers for Breast Cancer Subtypes

To evaluate the accuracy of the miRNAs as biomarkers for detection of breast cancer in serum, we determined the Receiver Operating Characteristic (ROC) curves, sensitivity and specificity of each miRNA. We considered an area under the ROC curve (AUC) ≥ 0.8 as a cutoff for further investigation and we identified 36 out of 42 differentially miRNAs as suitable biomarkers in the subtypes luminal A, luminal B, luminal B HER-2 positive and HER2-enriched (Table 2). For triple negative, miR-25-3p showed a slightly low AUC of 0.74.

**Figure 6:**
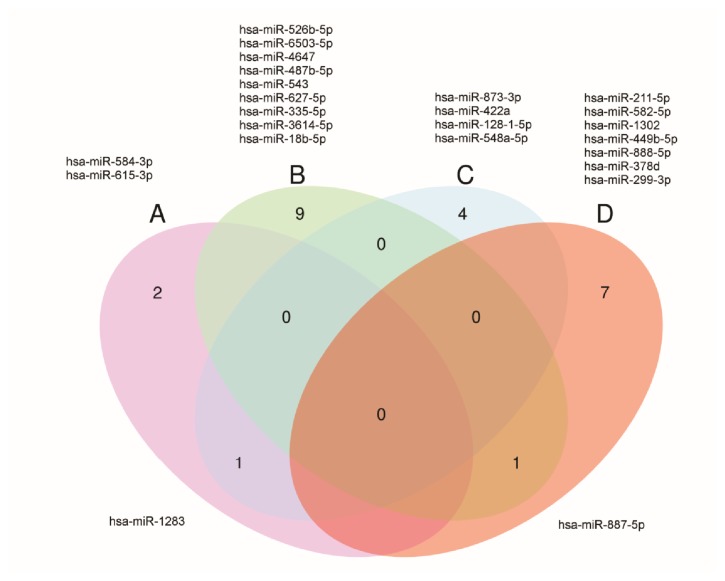
Venn diagram of downregulated miRNAs in serum of breast cancer patients. Venn diagram demonstrating 21 downregulated miRNAs, including miR-887-5p common between luminal A and luminal B HER2 positive. (a) HER 2; (b) luminal A; (c) luminal B; (d) luminal B HER2 positive.

**Figure 7:**
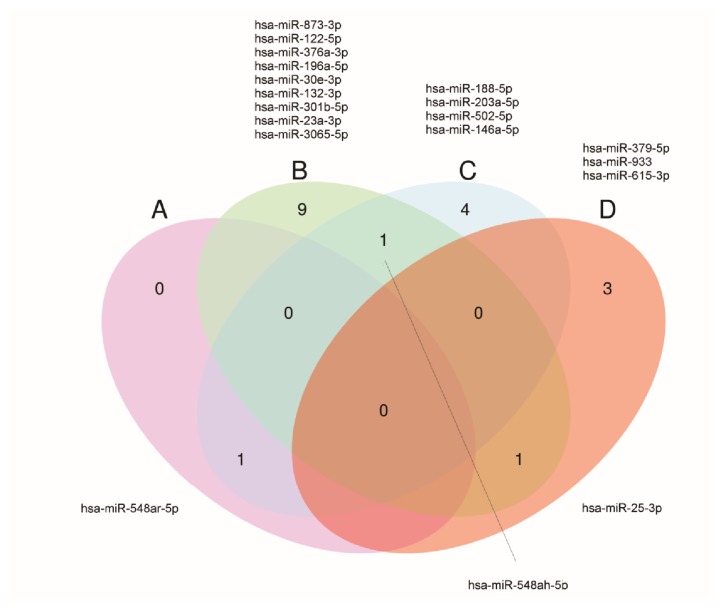
Venn diagram of upregulated miRNAs in serum of breast cancer patients. Venn diagram demonstrating 16 upregulated miRNAs, including miR-548ah-5p common between luminal A and luminal B, miR-548ar-5p common between HER2 and luminal B, and miR25-3p common between luminal A and luminal B HER 2 positive. (a) HER 2; (b) luminal A; (c) luminal B; (d) luminal B HER2 positive.

Among these 36 miRNAs, 21 were downregulated and 16 were upregulated with miR-615-3p being upregulated in luminal B HER 2 positive cases, but also being downregulated in HER2-enriched tumors (Figures 6 and 7). The subtype specificity of the 42 circulating miRNAs (Table 2) showed that 19 miRNAs were significantly differentially expressed in patients presenting with luminal A molecular subtype, 8 miRNAs in patients presenting with luminal B subtype, 10 miRNAs in patients presenting with luminal B HER 2 positive subtype, 4 miRNAs in patients presenting with the subtype in HER2-enriched, and only one miRNA in the triple negative subtype.

### 3.4. Functional* In Silico* Analysis

**Figure 8:**
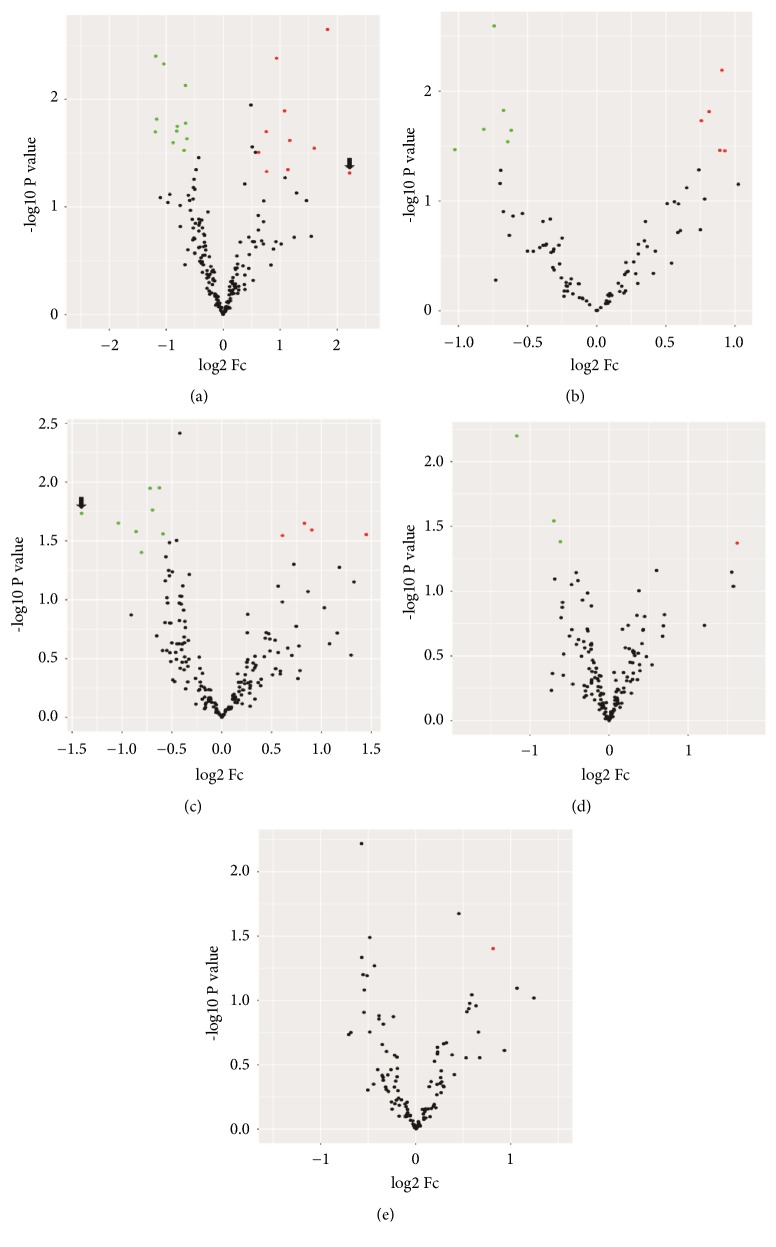
Differentially expressed miRNAs by molecular subtype breast cancer. Volcano plot demonstrating the profile of the differentially expressed miRNAs in different molecular subtypes of breast cancer. This plot demonstrates the fold change (x-axis) and the −log⁡10 P value (y-axis). The green circles represent the miRNAs downregulated and the red circles represent the miRNAs upregulated. The black circles indicate miRNAs that were not significantly expressed. Significance was determined with a P value cutoff of 0.05 and a 1.5-fold change. Molecular subtypes in breast cancer analyzed: (a) luminal A – arrow indicates the top upregulated miRNA (miR-25-3p); (b) luminal B; (c) luminal B HER 2 positive – arrow indicates the top downregulated miRNA (miR-378d); (d) HER2-enriched; (e) triple negative.

In order to identify the potential target mRNAs of the differentially expressed miRNAs, we identified the top increased (miR-25-3p, Fold change 3,56) and top decreased (miR-378d, Fold change: -2,65) miRNAs in each of the five molecular subtypes of breast cancer (Figure 8).

We identified the target genes for miR-378d and miR-25-3p using the online prediction tool miRDIP. We were unable to identify target genes predicted for miR-378d, but there were 14 target genes predicted for miR-25-3p.

Among these miR-25-3p predicted target genes, we found four molecular pathways (Integrin signalling pathway, EPHB forward signalling, FoxO signalling pathway and Ras signalling pathway) that had statistical significance for the regulation of cell adhesion, migration, and proliferation. These molecular pathways also overlap with the target gene NRAS see Figure S1 in the Supplementary Material for comprehensive image analysis).

## 4. Discussion

Mammographic screening is the gold-standard tool for the detection of early breast cancer lesions, yet it has several limitations such as false positive results and it is not very well accepted by all women since it is a very uncomfortable approach [17, 18]. In addition, breast cancer is classified in different histological and molecular subtypes, which present distinctive degrees of aggressiveness [17, 18]. Currently, only tissue samples obtained from conventional biopsy procedure can be useful for histological and molecular classification of this type of tumor. Thus, the liquid biopsy approach for breast cancer screening could improve the mammography sensitivity and could also be helpful for molecular classification [19, 20].

The miRNAs have attracted a great deal of attention as cancer biomarkers in the last few years due to the possibility of their detection from plasma or blood serum using conventional methods that could be adapted to clinical-laboratory routine [21, 22]. In addition, these circulating miRNAs have already been associated with the presence of various types of cancer [23, 24]. We therefore hypothesized that the identification of tumor-specific circulating miRNAs could be used for early detection of breast cancer as well as molecular biomarkers for identification of the different subtypes of breast cancer.

Most of minimally invasive biomarkers already described for breast cancer present low accuracy. A good example of minimally invasive biomarker for breast cancer is the CA 125, a serum biomarker that demonstrates 69% specificity and only 23% sensitivity [25]. Other serum biomarkers such as CEA and CA 15-3 are only reported to be effective in less than 15% of breast cancer patients [26].

An important finding of our study was that miR-25-3p could distinguish patients with triple negative breast cancer (the most aggressive subtype) from healthy controls. MiR-25-3p was also found to be upregulated in triple negative breast cancer tissue and cell lines [27]. Our prediction analyses showed that BTG2 is a putative target of miR-25-3p, so it seems possible that this miRNA may promote proliferation by targeting BTG2 in triple negative breast cancer. We found that miRNAs are important biomarkers for prognosis in triple negative breast cancer. For example, downregulated miR-221-3p is associated with poor prognostic biomarker for triple negative breast cancer [28]. However, few studies have been performed for early diagnosis using triple negative breast cancer because most cases of this aggressive molecular subtype are detected at an advanced stage.

In our study, it is possible that experimental variables due to sampling influenced the comparison between the 12 triple negative cases and the healthy controls. Also, the clinical and biological behavior of the triple negative molecular subtype is known to be more heterogeneous in comparison to the hormone receptor groups (luminal A, luminal B, luminal B HER 2 positive and HER2-enriched), which we were able to distinguish based on miRNA expression. Although the accuracy of the circulating miRNAs identified in HER2-enriched molecular subtype in our study was excellent, we believe it is necessary to validate these miRNAs using a larger cohort to increase the statistical power since only six patients were available for our analysis.

Considering that this study is retrospective, we have few serum breast cancer samples without any treatment available and small control group that Gail's Risk is determined. Thus, larger prospective studies are required to define the most robust circulating miRNA signature for improved clinical management of breast cancer using minimally invasive methods that avoid unnecessary biopsies. More extensive studies are needed to discover whether miR-25-3p could be a specific early detection biomarker for triple negative breast cancer.

## 5. Conclusions

Thus, in this case-control study we identified a molecular signature miRNAs as noninvasive biomarkers for each molecular subtype (luminal A, luminal B, luminal B HER2 positive and HER2-enriched breast) with increased precision. Future studies will be required to validate the clinical utility of these miRNAs using larger cohorts. Collectively our findings show that independently miRNAs can be detected in serum from patients with early breast cancer and their differential expression may be associated with specific molecular subtypes. Thus, the liquid biopsy approach using molecular biomarkers can be employed in the routine of breast cancer screening with potential to decrease the unnecessary invasive procedure.

## Figures and Tables

**Table 1:** Clinicopathological characteristics of the cases.

Characteristics	Value (n,%)
Age, years	
Median	54.6
Range	41-69
Molecular subtype	
Luminal A	12 (22.2%)
Luminal B	12 (22.2%)
Luminal B HER2 positive	12 (22.2%)
Triple negative	12 (22.2%)
HER 2+	6 (11.1%)
Stage, n	
Stage I	21 (38.9%)
Stage II	33 (61.1%)
Tumor size (TNM)	
T1	30 (55.6%)
T2	20 (37%)
T3	4 (7.4%)
Lymph node status (TNM)	
N0	33 (61.1%)
N1	21 (38.9%)
Histological type	
Ductal	44 (81.5%)
Others	10 (18.5%)

TNM classification of malignant tumors: T describes the tumor size of primary tumor; N describes regional lymph nodes that are involved; M describes distant metastasis.

**Table 2:** ROC curve of deregulated miRNAs by molecular subtype.

Molecular Subtype	miRNAs	Fc	Sensitivity	Specificity	Th	AUC
Luminal A	hsa-miR-18b-5p	-1.58	67%	83%	2.54	0.82
	hsa-miR-23a-3p	1.91	67%	100%	4.12	0.89
	hsa-miR-25-3p	3.56	92%	83%	3.08	0.92
	hsa-miR-487b-5p	-2.27	100%	92%	3.32	0.94
	hsa-miR-30e-3p	-2.05	100%	83%	2.85	0.92
	hsa-miR-122-5p	3.03	83%	83%	5.39	0.86
	hsa-miR-132-3p	-2.29	100%	75%	2.57	0.86
	hsa-miR-196a-5p	2.24	92%	83%	3.17	0.83
	hsa-miR-301b-5p	1.54	83%	83%	3.13	0.83
	hsa-miR-335-5p	1.69	75%	83%	2.75	0.85
	hsa-miR-376a-3p	2.20	83%	83%	3.53	0.83
	hsa-miR-526b-5p	-1.60	67%	83%	3.04	0.83
	hsa-miR-543	-1.84	67%	92%	3.64	0.89
	hsa-miR-548ah-5p	1.69	75%	83%	3.34	0.84
	hsa-miR-627-5p	-2.24	83%	92%	4.12	0.89
	hsa-miR-873-3p	2.11	100%	83%	3.13	0.90
	hsa-miR-887-5p	-1.75	83%	83%	3.21	0.87
	hsa-miR-3614-5p	-1.58	83%	75%	3.41	0.81
	hsa-miR-6503-5p	-1.76	67%	92%	3.33	0.83

Luminal B	hsa-miR-146a-5p	-1.67	83%	84%	3.20	0.83
	hsa-miR-188-5p	-1.60	83%	83%	3.79	0.83
	hsa-miR-203a-5p	1.87	75%	100%	2.64	0.80
	hsa-miR-502-5p	1.69	75%	100%	2.91	0.83
	hsa-miR-548ar-5p	1.90	67%	100%	5.05	0.81
	hsa-miR-548a-5p	1.85	83%	83%	2.71	0.85
	hsa-miR-548ah-5p	1.75	67%	83%	3.69	0.81
	hsa-miR-128-1-5p	-1.53	100%	75%	2.64	0.80

Luminal B HER2+	hsa-miR-25-3p	2.73	75%	83%	3.32	0.82
	hsa-miR-378d	-2.65	67%	92%	4.31	0.83
	hsa-miR-379-5p	1.87	67%	83%	3.13	0.85
	hsa-miR-449b-5p	-1.65	67%	83%	3.10	0.83
	hsa-miR-582-5p	-2.05	67%	75%	3.84	0.80
	hsa-miR-615-3p	1.78	67%	100%	2.96	0.83
	hsa-miR-887-5p	-1.74	67%	92%	3.71	0.82
	hsa-miR-888-5p	-1.62	83%	75%	3.38	0.86
	hsa-miR-933	1.53	83%	83%	2.47	0.89
	hsa-miR-1302	-1.54	83%	75%	3.27	0.80

HER2+	hsa-miR-548ar-5p	3.07	100%	77%	5.24	0.97
	hsa-miR-584-3p	-1.53	100%	100%	4.45	1.00
	hsa-miR-615-3p	-1.62	100%	84%	4.20	0.94
	hsa-miR-1283	-2.24	100%	100%	6.09	1.00

Accuracy of deregulated miRNAs with ROC curve ≥ 0.8. Fc: fold-change; Th: threshold; AUC: area under the curve.

## Data Availability

The functional analyses and differentially expressed miRNAs data used to support the findings of this study are available from the corresponding author upon request.

## Supplementary Materials

Supplementary Materials Table S1: miR-25-3p target genes predicted and molecular pathways determined by Reactome.
